# G Protein-Coupled Receptor Kinases Take Central Stage

**DOI:** 10.3390/cells12010023

**Published:** 2022-12-21

**Authors:** Federico Mayor, Cristina Murga

**Affiliations:** 1 Departamento de Biología Molecular, Instituto Universitario de Biología Molecular (IUBM) and Centro de Biología Molecular Severo Ochoa (CBMSO), Universidad Autónoma de Madrid, 28049 Madrid, Spain; 2 Instituto de Investigación Sanitaria Hospital Universitario La Princesa, 28006 Madrid, Spain; 3 CIBER Cardiovascular (CIBERCV), Instituto de Salud Carlos III (ISCIII), 28035 Madrid, Spain

The relevance of the family of G protein-coupled receptor kinases (GRKs) is based on its key participation in the regulation and intracellular dynamics of the largest family of membrane receptors, namely G protein-coupled receptors (GPCRs). The phosphorylation of GPCRs by GRKs at serine/threonine residues triggers the association of regulatory β-arrestins proteins that prompt the uncoupling and termination (desensitization) of G protein-dependent cascades, but also receptor internalization and re-sensitization processes that are essential for profiling signal transduction by GPCRs. GRK-dependent recruitment of β-arrestins to GPCRs also initiates the downstream arrestin-dependent cascades that are one of the hallmarks of integrative signalling downstream of these receptors [1]. Furthermore, GRKs are currently considered veritable signal transducers themselves, since they can also phosphorylate and/or functionally interact with a wide variety of non-GPCR proteins [2]. Therefore, the seven GRKs, and, in particular, the ubiquitous and essential GRK2 isoform, are nowadays regarded as key cell-signal regulators that modulate intracellular crosstalk among signalling routes and also the fine tuning of threshold response levels in many cell types and tissues.

Through such complex interactomes, GRKs and arrestins are able to impact the onset, prognosis, diagnosis or development of many important human pathologies such as neurological, cardiovascular, tumoral or metabolic disorders among others. Coherently, changes in the levels or functionality of GRKs are increasingly detected in different pathological contexts both in humans and animal models of disease, and regarded as potential targets for the design of disease modifiers and novel treatments [2,3,4,5].

The eleven articles and reviews in this Special Issue of *Cells*, contributed by leading laboratories in this research area, seek to unravel some of the most innovative insights into the molecular and cellular biology of GRKs with particular emphasis on their impact on human pathophysiology.

Jeff Benovic, who pioneered the discovery of this family of proteins in the laboratory of Prof. Robert J. Lefkowitz, provided a historical perspective of research on GRKs since the identification, purification and cloning of the β-adrenergic receptor kinase (later renamed GRK2) in the 1980s [6]. For an update on the role of GRKs in the regulation of neuronal signalling, Eugenia and Vsevolod Gurevich resourcefully revisit novel concepts in the regulation by GRKs of different neurotransmitter receptors, with emphasis on GPCR oligomerization, biased signaling, phosphorylation barcodes, arrestin recruitment, and on the features and kinetics of desensitization and internalization/resensitization cycles [7]. In this line, the group of Meritxell Canals reviews our current understanding of the molecular determinants and mechanisms of opioid receptor phosphorylation by GRKs and its impact on the actions of analgesic drugs and opioid-induced tolerance and addiction, with a particular focus on the mu isoform of the opioid receptor (MOR) as a target of widely used drugs such as morphine or fentanyl [8].

The important role of GRKs in immune cells and in the regulation of inflammatory signals is also covered in this Special Issue. The group of Françoise Bachelerie [9] describes the contribution of GRKs to the functionality and migration of immune cells in response to chemokine gradients, highlighting the importance of receptor phosphorylation barcodes. They also dwell on alterations in neutrophil migration patterns based on changes in GRKs or GPCRs levels/functionality under pathological conditions. Related research by the Martine J Smit laboratory deepens understanding of the cell biology of the atypical chemokine receptor 3 (ACKR3) using various bioluminescence and fluorescence resonance energy transfer-based sensors to measure the CXCL12-induced recruitment of GRKs and arrestins. This study unveils differences in the requirements of different GRK and arrestin isoforms for ACKR3 modulation, and also identifies the C-terminal tail residues required for internalization [10]. Additionally, Linda M. McAllister-Lucas and collaborators review the impact of GRK′s canonical and noncanonical roles on the normal immune cell function of T and B lymphocytes, and potential implications on the molecular pathogenesis of inflammatory and neoplastic disease [11]. These new data and insights are particularly timely given the emerging relevant role of chemokine receptors and GRKs in different cell types in the tumor microenvironment [5,12,13]. In this context, the work of Petronila Penela and collaborators [14] put forward a surprising novel function of GRK2 in centrosome dynamics and mitotic spindle functionality, involving complex functional interactions among GRK2, the Hippo pathway component MST2 and the E3 ligase Mdm2.

In relation to cardiovascular and metabolic function, the group of Giuseppe Rengo reviews current information regarding the possible use of lymphocyte GRK2 levels as a surrogate marker of hyperactivation of the cardiac adrenergic nervous system as a hallmark of heart failure in humans that may provide independent prognostic information for the improvement of other currently available techniques [15]. In addition, Guido Iaccarino and collaborators analyze current knowledge on the metabolic role of GRK2 in conditions related to insulin resistance such as obesity, hypertension or glucose intolerance and the potential of inhibiting GRK2 as a therapeutic strategy in cardiovascular or metabolic diseases [16]. In this context, a review by the group of Frank Lezoualc’h summarizes the pathophysiological roles of GRK isoforms and Epac1 (exchange protein directly activated by cAMP 1) in the heart, focusing on how these proteins cross-talk in nodal signalosomes that contribute to impaired cardiac function and tissue remodelling during stress conditions [17]. Finally, the work of Arcones et al. shows that changes in GRK2 protein levels in murine hearts display sexual dimorphism, with lower GRK2 amounts in young female mice, and cardiac GRK2 upregulation taking place with age only in females and also upon ovariectomy. The fact that ovarian hormones regulate GRK2 levels in the heart may have potential implications in the sex-dependent dynamics of cardiovascular disease risk, particularly in post-menopausal women [18].

Overall, this Special Issue provides a comprehensive update about the emerging roles and physio-pathological implications of altered GRKs levels and functions, which may help to foster new collaborative research in this area to better understand the role of GRKs in the maladaptive rewiring of signalling networks taking place in different disease contexts and to design new treatment strategies.
